# Supplementary material for: Structural basis for emetine inhibition of ribosome translocation in Toxoplasma gondii
Source: bioRxiv. 2026 Jul 24:2026.07.23.740346. Preprint. [Version 1] doi: 10.64898/2026.07.23.740346 (PMC13419753; doi:10.64898/2026.07.23.740346)
Supplement: Supplement 1 [file NIHPP2026.07.23.740346v1-supplement-1.pdf]

bioRxiv preprint doi: https://doi.org/10.64898/2026.07.23.740346; this version posted July 24, 2026. The copyright holder for this preprint (which was not certified by peer review) is the author/funder, who has granted bioRxiv a license to display the preprint in perpetuity. It is made available under a CC-BY 4.0 International license.

Structural basis for emetine inhibition of ribosome translocation in *Toxoplasma gondii*

Wenzhao Dong[1,2,3*], Fengrong Wang[1,2,3,4,5*], Brenna A. Saladin[1,2,3], Suada Leskaj[1,2,3], Ritam Neupane[1,2,3], Vern Carruthers[4#], Jailson Brito Querido[1,2,3#]

(1) Department of Biological Chemistry, University of Michigan, Ann Arbor, MI 48109, USA
(2) Life Sciences Institute, University of Michigan, Ann Arbor, MI 48109, USA
(3) Center for RNA Biomedicine, University of Michigan, Ann Arbor, MI 48109, USA
(4) Department of Microbiology and Immunology, University of Michigan Medical School, Ann Arbor, MI 48109, USA.
(5) Department of Biomedical Sciences, Iowa State University, Ames, IA 50010, USA

* These authors contributed equally
[#]To whom correspondence should be addressed: vcarruth@umich.edu & jquerido@umich.edu

Supplemental figures and tables

Supplemental Figure 1: Purification workflow for actively translating *T. gondii* 80S ribosome complexes for cryoEM and mass spectrometry analyses. *T. gondii* parasites were cultured in human fibroblasts and purified prior to cell lysis. Emetine was added to stall actively translating ribosomes on mRNAs and preserve translation complexes. Cell lysates were subjected to sucrose density gradient ultracentrifugation to separate ribosomal species, including free mRNAs, 40S/43S/48S particles, 60S subunits, 80S monosomes, and polysomes. Fractions corresponding to 80S ribosome complexes were collected from the sucrose gradient and used for cryoEM and mass spectrometry analyses.

Supplemental Figure 2: Sequence conservation of RACK1 and uS3 among *T. gondii*, *P. falciparum*, human, and yeast. **(A)**. Multiple sequence alignment of RACK1 proteins from *T. gondii, P. falciparum*, human and yeast. Conserved residues are highlighted with darker shading indicating higher sequence conservation. Alignments were generated using Muscle with defaults, Jalview. **(B)**. Percent identity matrix showing pairwise amino acid sequence identities *T. gondii, P. falciparum* and human. **(C)**. Multiple sequence alignment of uS3 proteins from *T. gondii, P. falciparum* and human. Conserved residues are highlighted with darker shading indicating higher sequence conservation. Alignments were generated using Muscle with defaults, Jalview.

bioRxiv preprint doi: https://doi.org/10.64898/2026.07.23.740346; this version posted July 24, 2026. The copyright holder for this preprint (which was not certified by peer review) is the author/funder, who has granted bioRxiv a license to display the preprint in perpetuity. It is made available under a CC-BY 4.0 International license.

Supplemental Figure 3: Sequence alignment of *H. sapiens* SERBP1 and Putative *T. gondii* SERBP1. Alignments were generated using Muscle with defaults, Jalview.

Supplemental Figure 4: *T. gondii* 80S RACK1 local resolution information of postprocess maps and atomic models. **(A).** Final resolution/FSC curve of density maps for 40S head, 40S body and 60S respectively; **(B).** local resolution information of density maps for 40S head, 40S body and 60S respectively; **(C).** Final resolution/FSC curve of atomic models for 40S head, 40S body and 60S respectively

Supplemental Figure 5: *T. gondii* 80S PRE-H1 local resolution information of postprocess maps and atomic models. **(A).** Final resolution/FSC curve of density maps for 40S head, 40S body and 60S respectively; **(B).** local resolution information of density maps for 40S head, 40S body and 60S respectively; **(C).** Final resolution/FSC curve of atomic models for 40S head, 40S body and 60S respectively.

Supplemental Figure 6: *T. gondii* 80S PRE-H2 local resolution information of postprocess maps and atomic models. **(A).** Final resolution/FSC curve of density maps for 40S head, 40S body and 60S respectively; **(B).** local resolution information of density maps for 40S head, 40S body and 60S respectively; **(C).** Final resolution/FSC curve of atomic models for 40S head, 40S body and 60S respectively.

Supplemental Figure 7: *T. gondii* 80S eEF2 local resolution information of postprocess maps and atomic models. **(A).** Final resolution/FSC curve of density maps for 40S head, 40S body and 60S respectively; **(B).** local resolution information of density maps for 40S head, 40S body and 60S respectively; **(C).** Final resolution/FSC curve of atomic models for 40S head, 40S body and 60S respectively.

Supplemental Figure 8: *T. gondii* 80S Emetine Class1 local resolution information of postprocess maps and atomic models. **(A).** Final resolution/FSC curve of density maps for 40S head, 40S body and 60S respectively; **(B).** local resolution information of density maps for 40S head, 40S body and 60S respectively; **(C).** Final resolution/FSC curve of atomic models for 40S head, 40S body and 60S respectively

Supplemental Figure 9: *T. gondii* 80S Emetine Class2 local resolution information of postprocess

bioRxiv preprint doi: https://doi.org/10.64898/2026.07.23.740346; this version posted July 24, 2026. The copyright holder for this preprint (which was not certified by peer review) is the author/funder, who has granted bioRxiv a license to display the preprint in perpetuity. It is made available under a CC-BY 4.0 International license.

maps and atomic models. **(A).** Final resolution/FSC curve of density maps for 40S head, 40S body and 60S respectively; **(B).** local resolution information of density maps for 40S head, 40S body and 60S respectively; **(C).** Final resolution/FSC curve of atomic models for 40S head, 40S body and 60S respectively

Supplemental Figure 10: Human 80S local resolution information of postprocess maps and atomic Models. **(A).** Final resolution/FSC curve of density maps for 40S head, 40S body and 60S respectively; **(B).** local resolution information of density maps for 40S head, 40S body and 60S respectively; **(C).** Final resolution/FSC curve of atomic models for 40S head, 40S body and 60S respectively

Supplemental Figure 11: *T. gondii* 80S data process flow chart

Supplemental Figure 12: Human 80S data process flow chart

Supplementary Table 1. Mass spectrometry analysis of purified *T. gondii* 80S ribosome complexes
LC–MS/MS analysis of proteins identified in purified *T. gondii* 80S ribosome complexes. The table includes identified proteins and peptides, sequence coverage, peptide-spectrum matches (PSMs), quantitative abundance values, and functional annotations generated from proteomic analysis.

Supplementary Table 2. Cryo-EM data collection, refinement and validation statistics

bioRxiv preprint doi: https://doi.org/10.64898/2026.07.23.740346; this version posted July 24, 2026. The copyright holder for this preprint (which was not certified by peer review) is the author/funder, who has granted bioRxiv a license to display the preprint in perpetuity. It is made available under a CC-BY 4.0 International license.

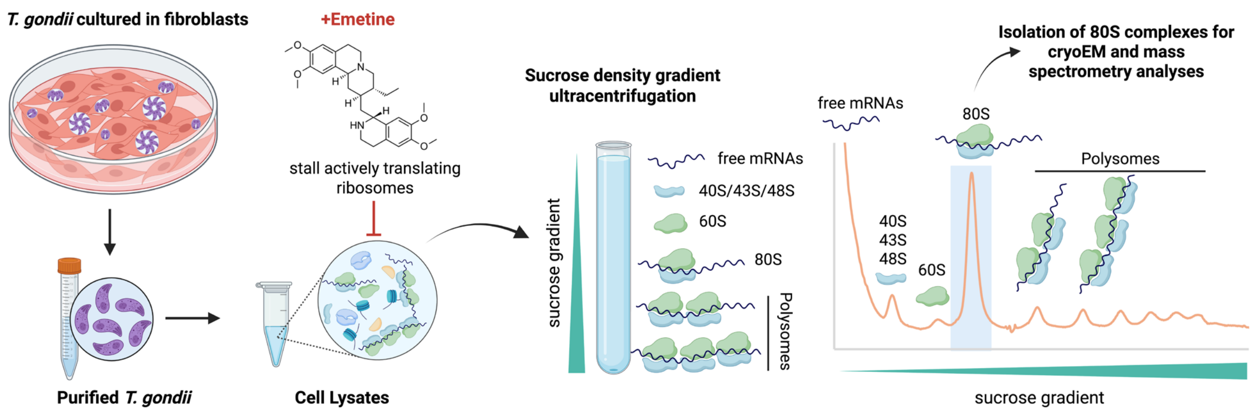

bioRxiv preprint doi: https://doi.org/10.64898/2026.07.23.740346; this version posted July 24, 2026. The copyright holder for this preprint (which was not certified by peer review) is the author/funder, who has granted bioRxiv a license to display the preprint in perpetuity. It is made available under a CC-BY 4.0 International license.

A RACK1 alignment among *T. gondii, P. falciparum*, *H. sapiens* and *S. cerevisiae*

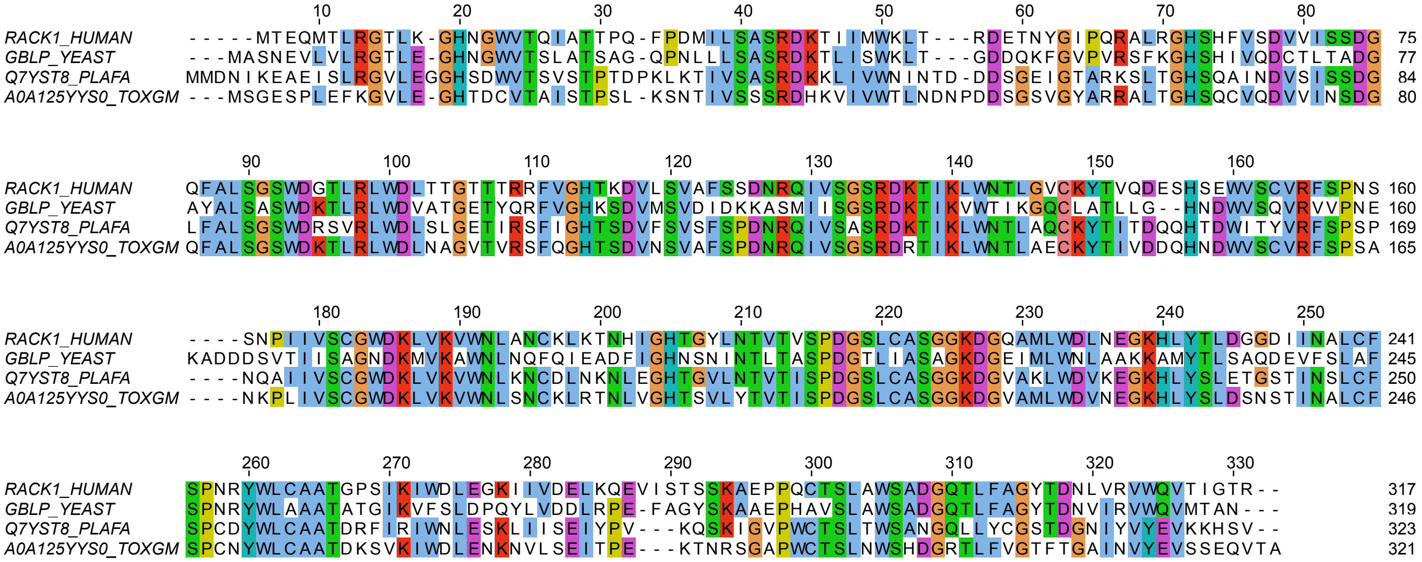

B Percent identity matrix of RACK1 alignment among *T. gondii, P. falciparum* and *H. sapiens*

	H. sapiens	*P. falciparum*	*T. gondii*
H. sapiens	100.00%	58.79%	61.15%
P. falciparum	58.79%	100.00%	67.30%
T. gondii	61.15%	67.30%	100%

C uS3 alignment among *T. gondii, P. falciparum* and *H. sapiens*

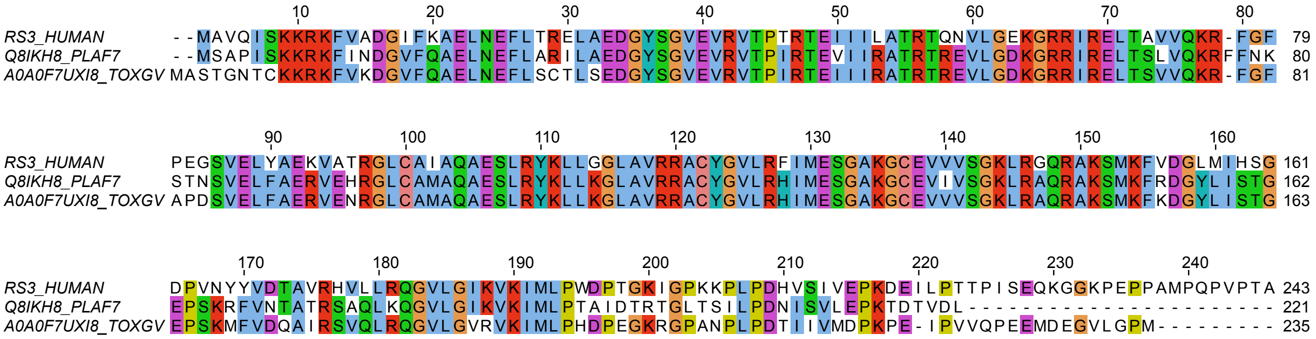

bioRxiv preprint doi: https://doi.org/10.64898/2026.07.23.740346; this version posted July 24, 2026. The copyright holder for this preprint (which was not certified by peer review) is the author/funder, who has granted bioRxiv a license to display the preprint in perpetuity. It is made available under a CC-BY 4.0 International license.

A

Sequence alignment of *H. sapiens* SERBP1 and Putative *T. gondii* SERBP1

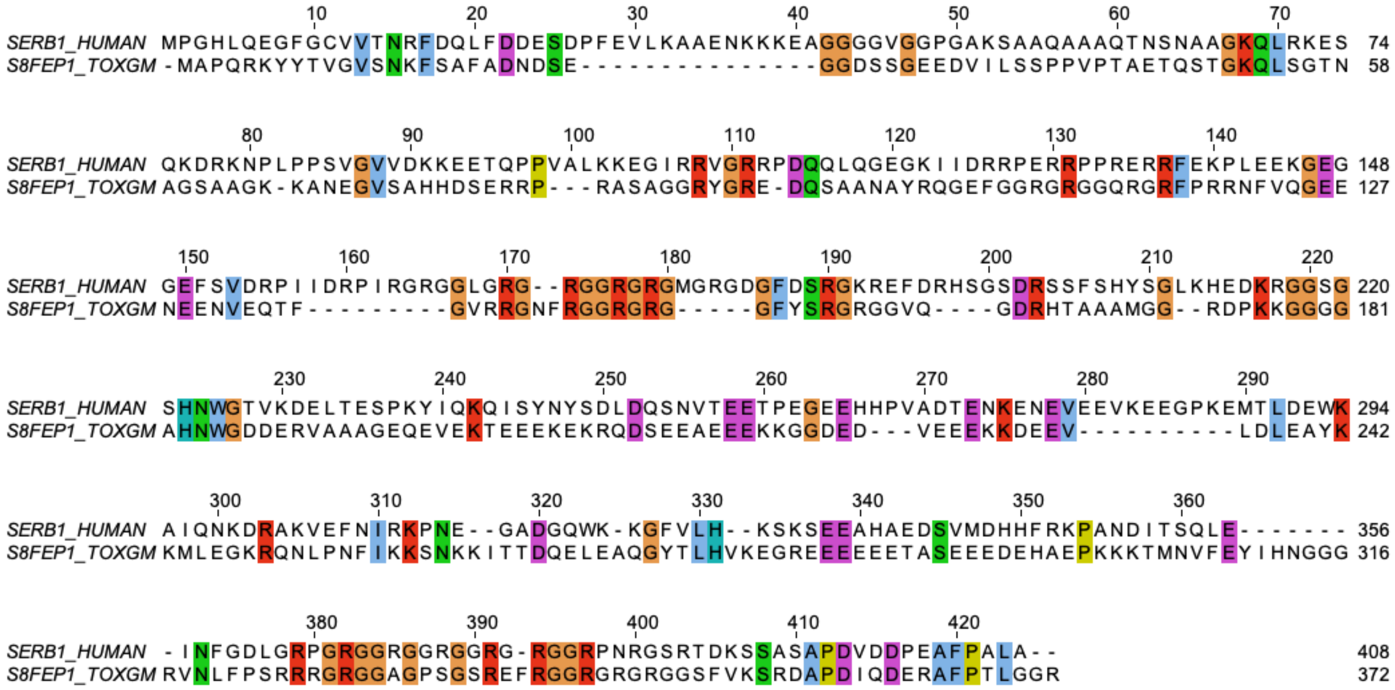

bioRxiv preprint doi: https://doi.org/10.64898/2026.07.23.740346; this version posted July 24, 2026. The copyright holder for this preprint (which was not certified by peer review) is the author/funder, who has granted bioRxiv a license to display the preprint in perpetuity. It is made available under a CC-BY 4.0 International license.

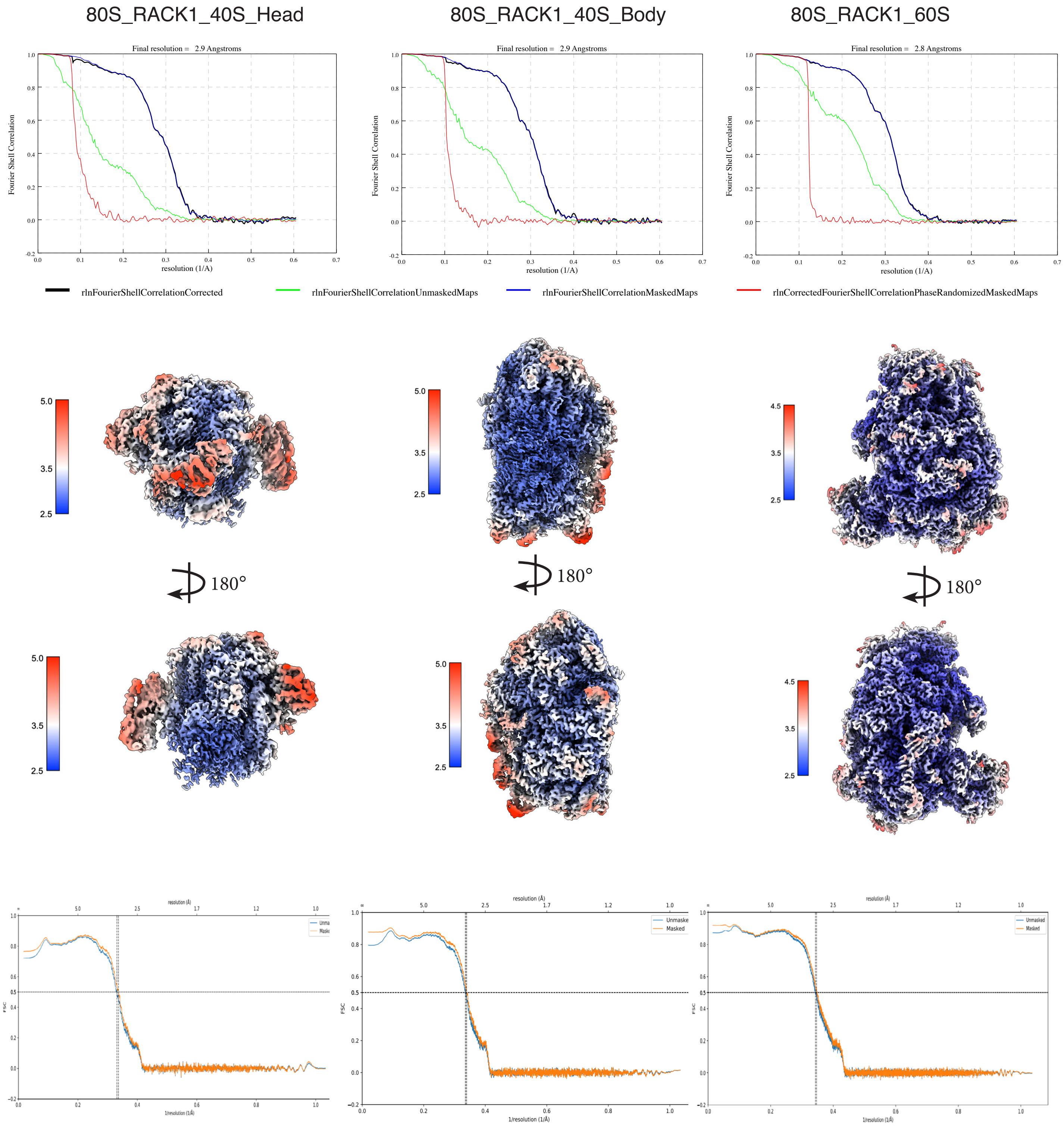

bioRxiv preprint doi: https://doi.org/10.64898/2026.07.23.740346; this version posted July 24, 2026. The copyright holder for this preprint (which was not certified by peer review) is the author/funder, who has granted bioRxiv a license to display the preprint in perpetuity. It is made available under a CC-BY 4.0 International license.

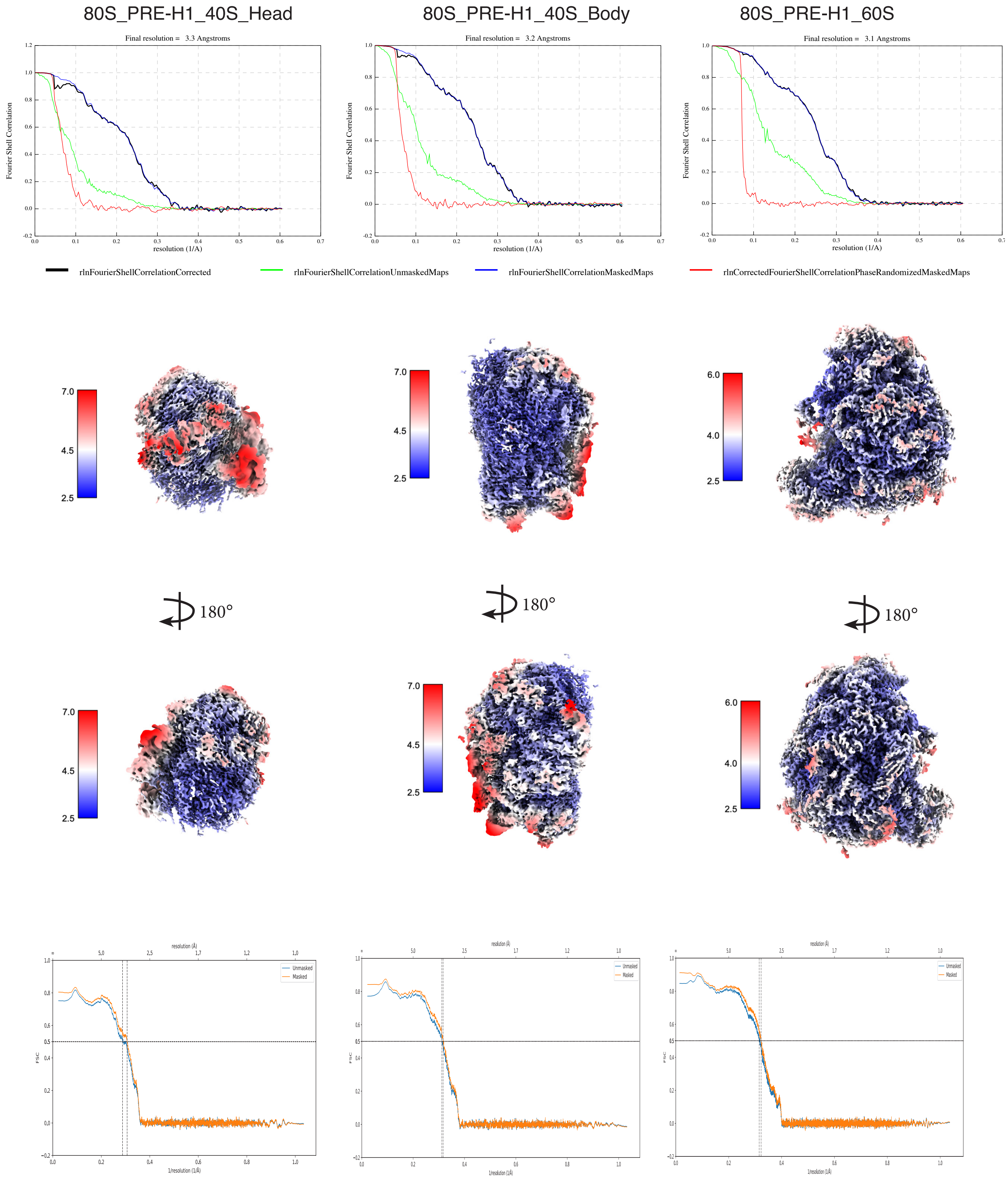

bioRxiv preprint doi: https://doi.org/10.64898/2026.07.23.740346; this version posted July 24, 2026. The copyright holder for this preprint (which was not certified by peer review) is the author/funder, who has granted bioRxiv a license to display the preprint in perpetuity. It is made available under a CC-BY 4.0 International license.

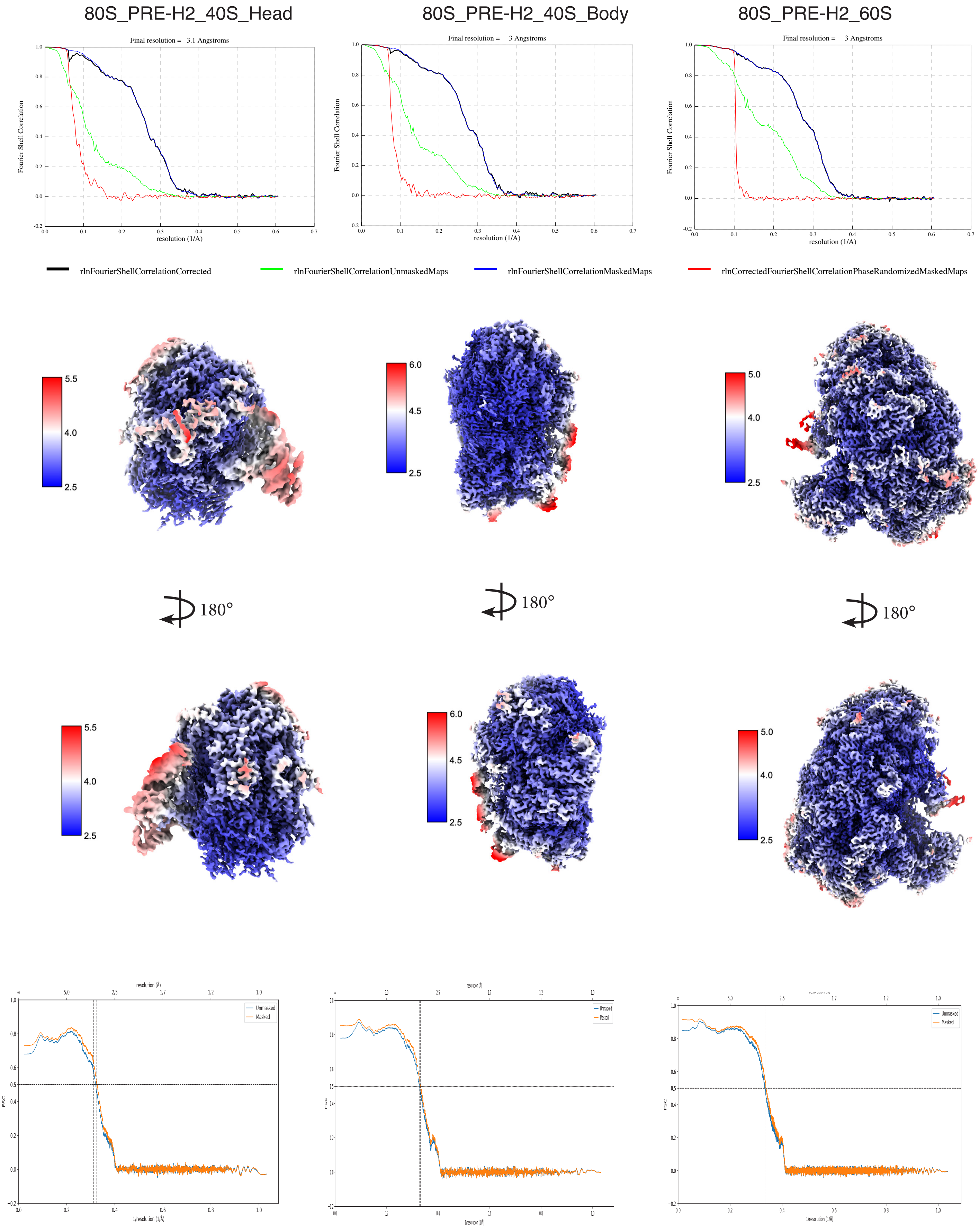

bioRxiv preprint doi: https://doi.org/10.64898/2026.07.23.740346; this version posted July 24, 2026. The copyright holder for this preprint (which was not certified by peer review) is the author/funder, who has granted bioRxiv a license to display the preprint in perpetuity. It is made available under a CC-BY 4.0 International license.

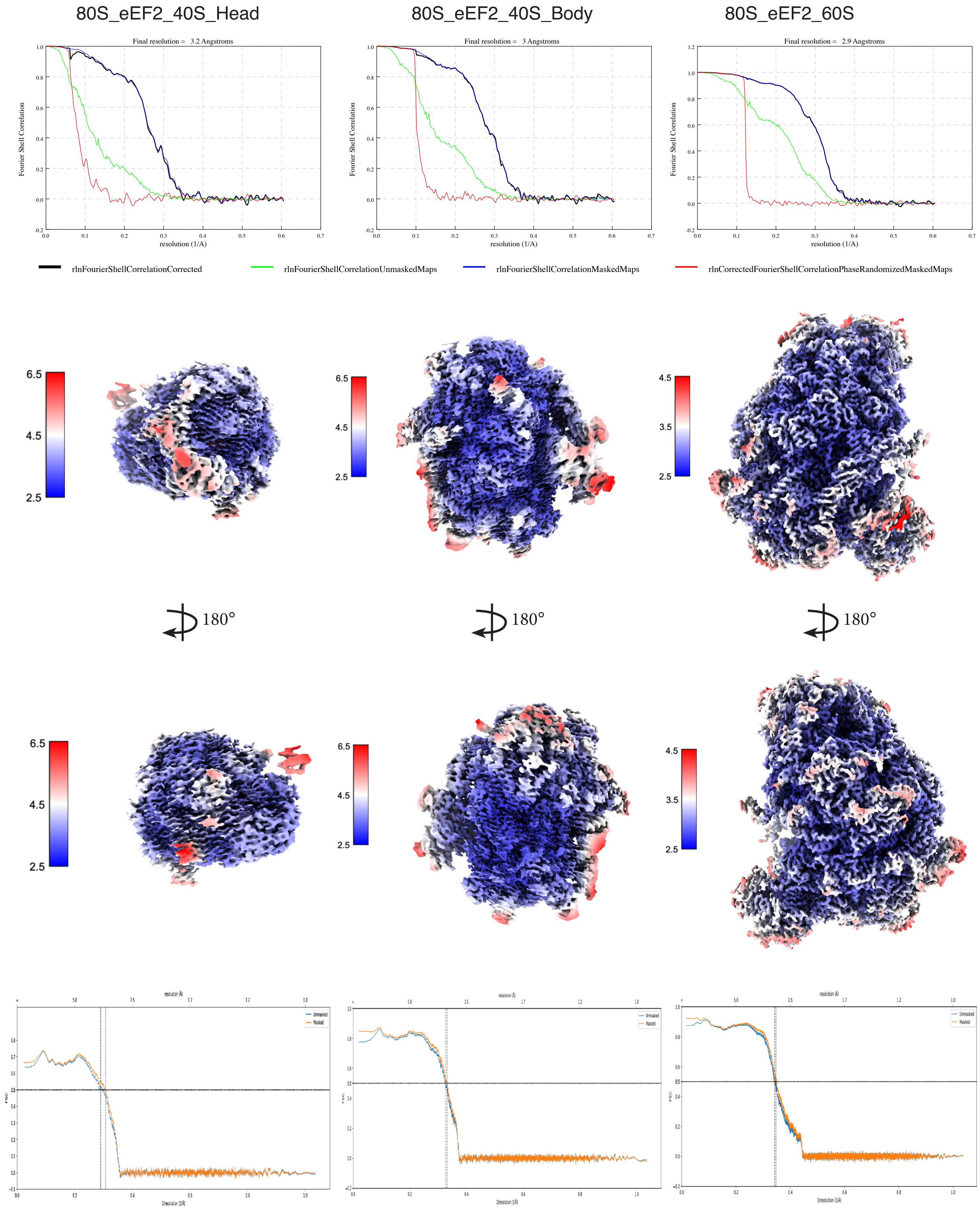

bioRxiv preprint doi: https://doi.org/10.64898/2026.07.23.740346; this version posted July 24, 2026. The copyright holder for this preprint (which was not certified by peer review) is the author/funder, who has granted bioRxiv a license to display the preprint in perpetuity. It is made available under a CC-BY 4.0 International license.

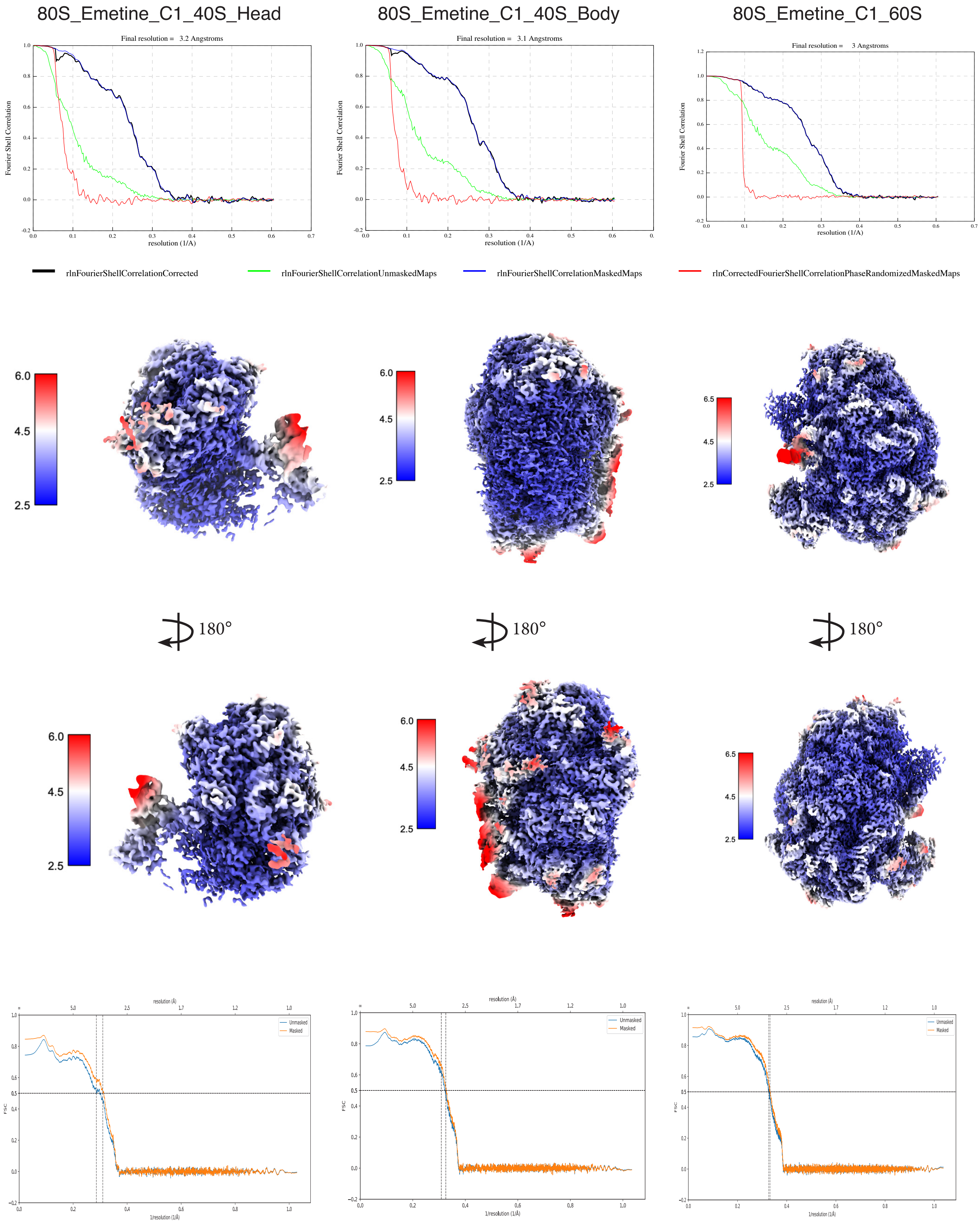

bioRxiv preprint doi: https://doi.org/10.64898/2026.07.23.740346; this version posted July 24, 2026. The copyright holder for this preprint (which was not certified by peer review) is the author/funder, who has granted bioRxiv a license to display the preprint in perpetuity. It is made available under a CC-BY 4.0 International license.

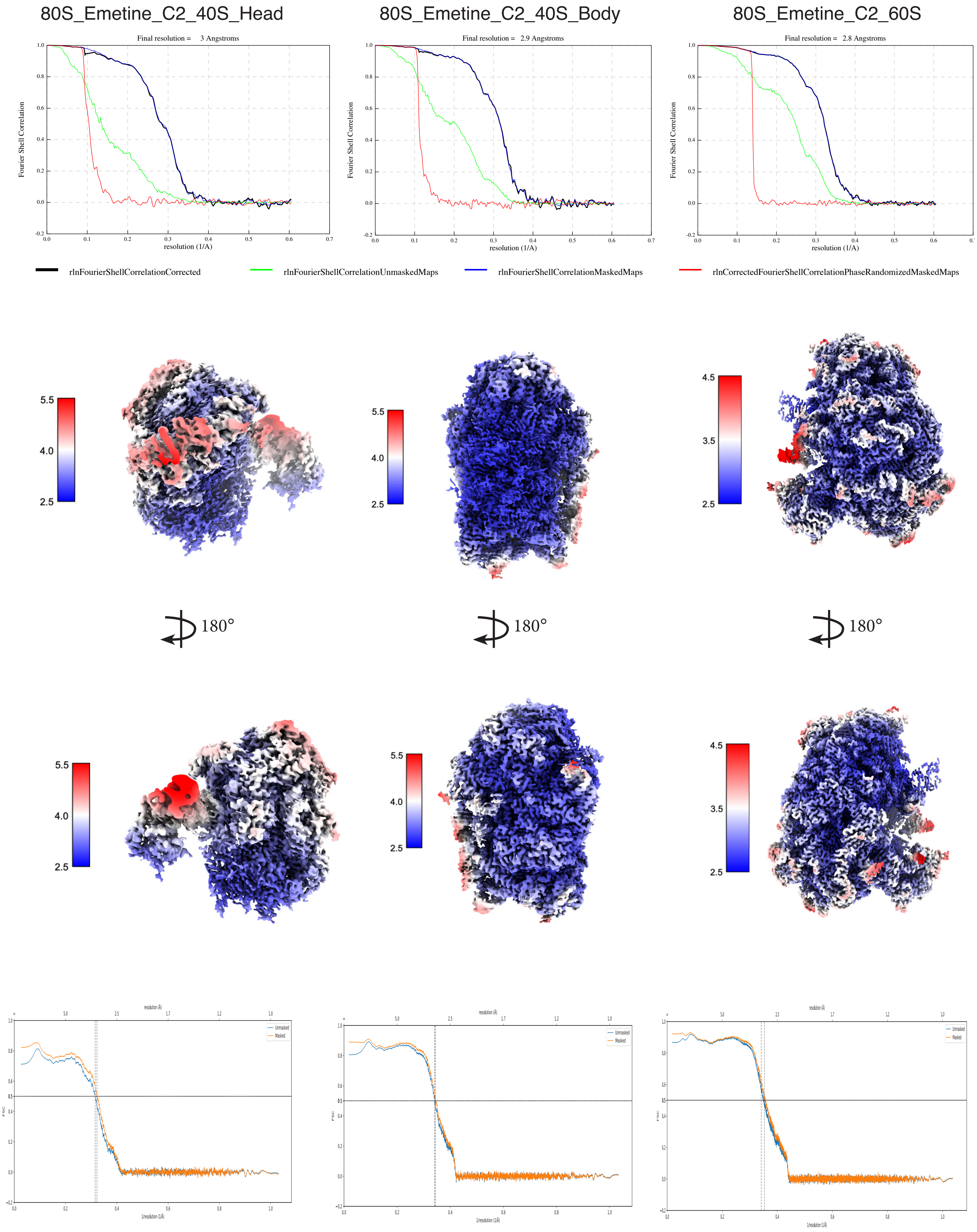

bioRxiv preprint doi: https://doi.org/10.64898/2026.07.23.740346; this version posted July 24, 2026. The copyright holder for this preprint (which was not certified by peer review) is the author/funder, who has granted bioRxiv a license to display the preprint in perpetuity. It is made available under a CC-BY 4.0 International license.

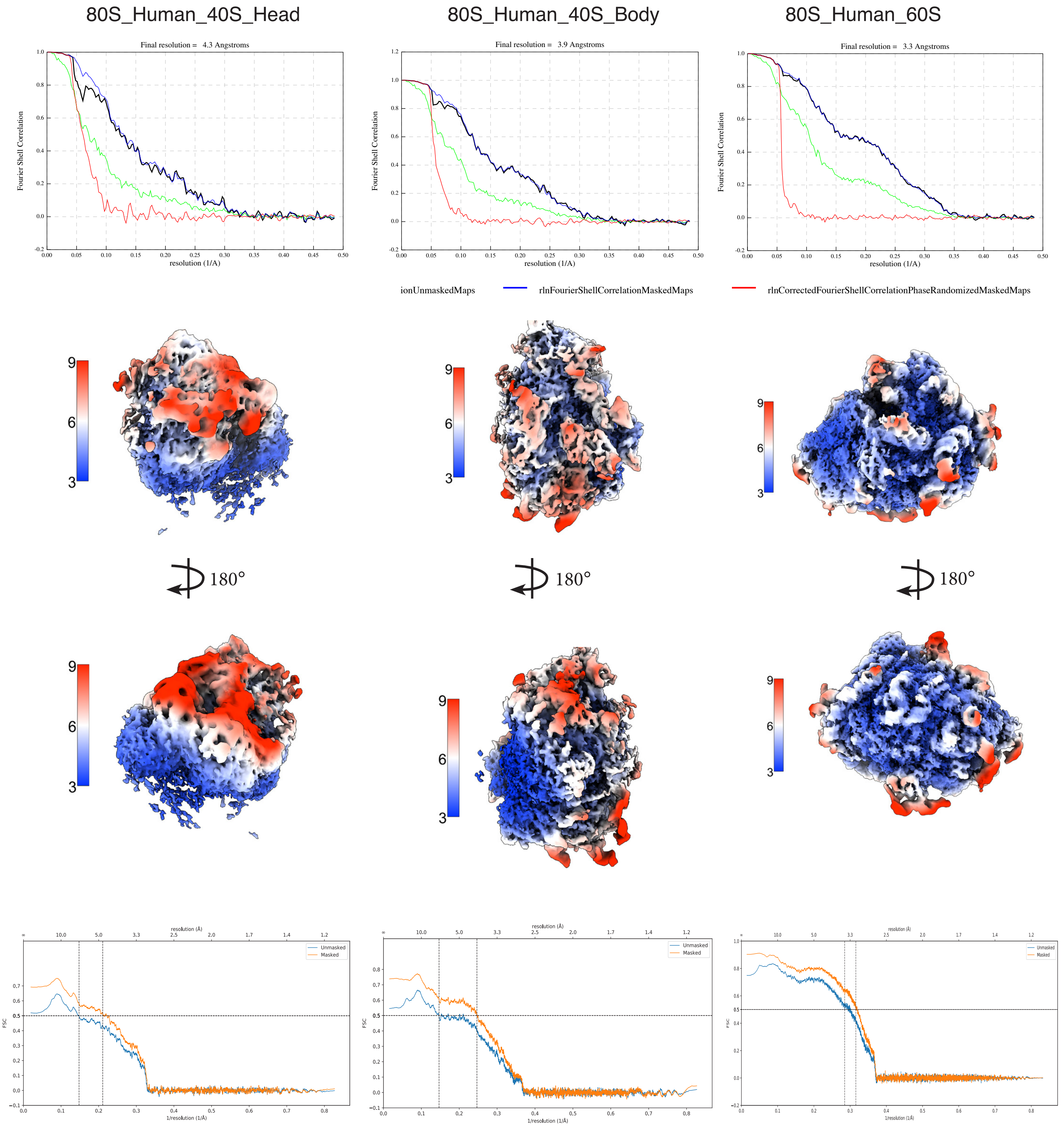

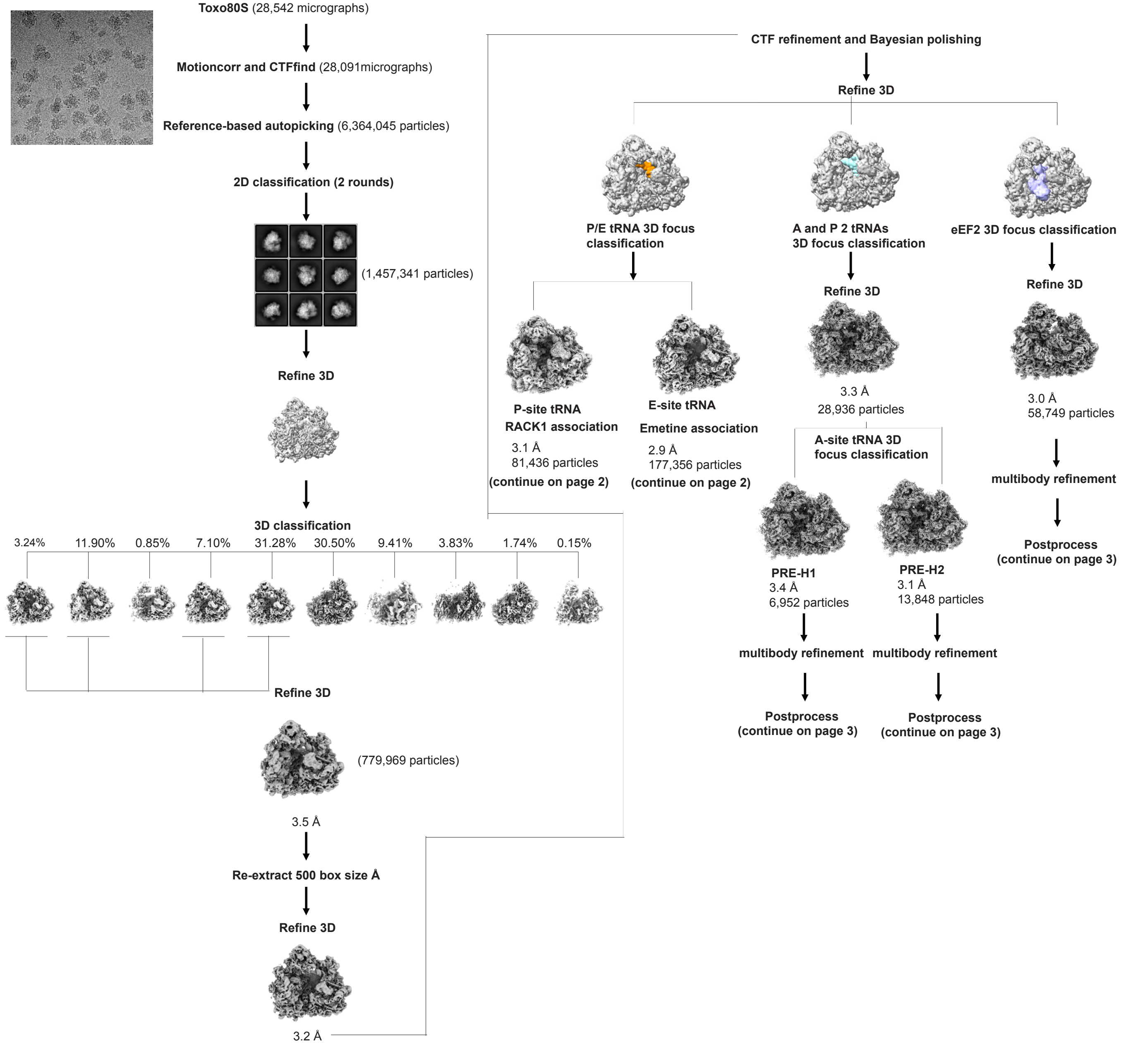
Toxo80S (28,542 micrographs)
Motioncorr and CTFfind (28,091micrographs)
Reference-based autopicking (6,364,045 particles)
2D classification (2 rounds)
(1,457,341 particles)
Refine 3D
3D classification
3.24%
11.90%
0.85%
7.10%
31.28%
30.50%
9.41%
3.83%
1.74%
0.15%
Refine 3D
(779,969 particles)
3.5 Å
Re-extract 500 box size Å
Refine 3D
3.2 Å
CTF refinement and Bayesian polishing
Refine 3D
P/E tRNA 3D focus classification
A and P 2 tRNAs 3D focus classification
eEF2 3D focus classification
P-site tRNA
RACK1 association
3.1 Å
81,436 particles
(continue on page 2)
E-site tRNA
Emetine association
2.9 Å
177,356 particles
(continue on page 2)
Refine 3D
3.3 Å
28,936 particles
A-site tRNA 3D focus classification
PRE-H1
3.4 Å
6,952 particles
multibody refinement
Postprocess
(continue on page 3)
PRE-H2
3.1 Å
13,848 particles
multibody refinement
Postprocess
(continue on page 3)
Refine 3D
3.0 Å
58,749 particles
multibody refinement
Postprocess
(continue on page 3)

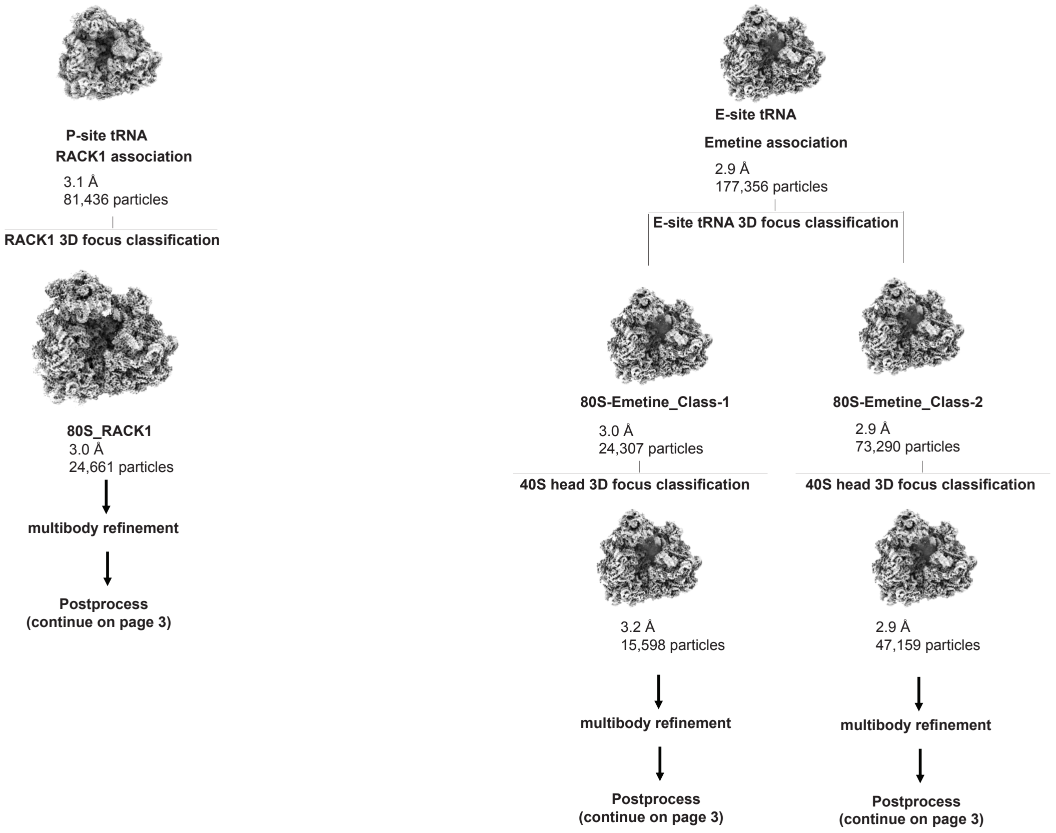

P-site tRNA
RACK1 association
3.1 Å
81,436 particles
RACK1 3D focus classification
80S_RACK1
3.0 Å
24,661 particles
multibody refinement
Postprocess
(continue on page 3)
E-site tRNA
Emetine association
2.9 Å
177,356 particles
E-site tRNA 3D focus classification
80S-Emetine_Class-1
3.0 Å
24,307 particles
80S-Emetine_Class-2
2.9 Å
73,290 particles
40S head 3D focus classification
40S head 3D focus classification
3.2 Å
15,598 particles
2.9 Å
47,159 particles
multibody refinement
multibody refinement
Postprocess
(continue on page 3)
Postprocess
(continue on page 3)

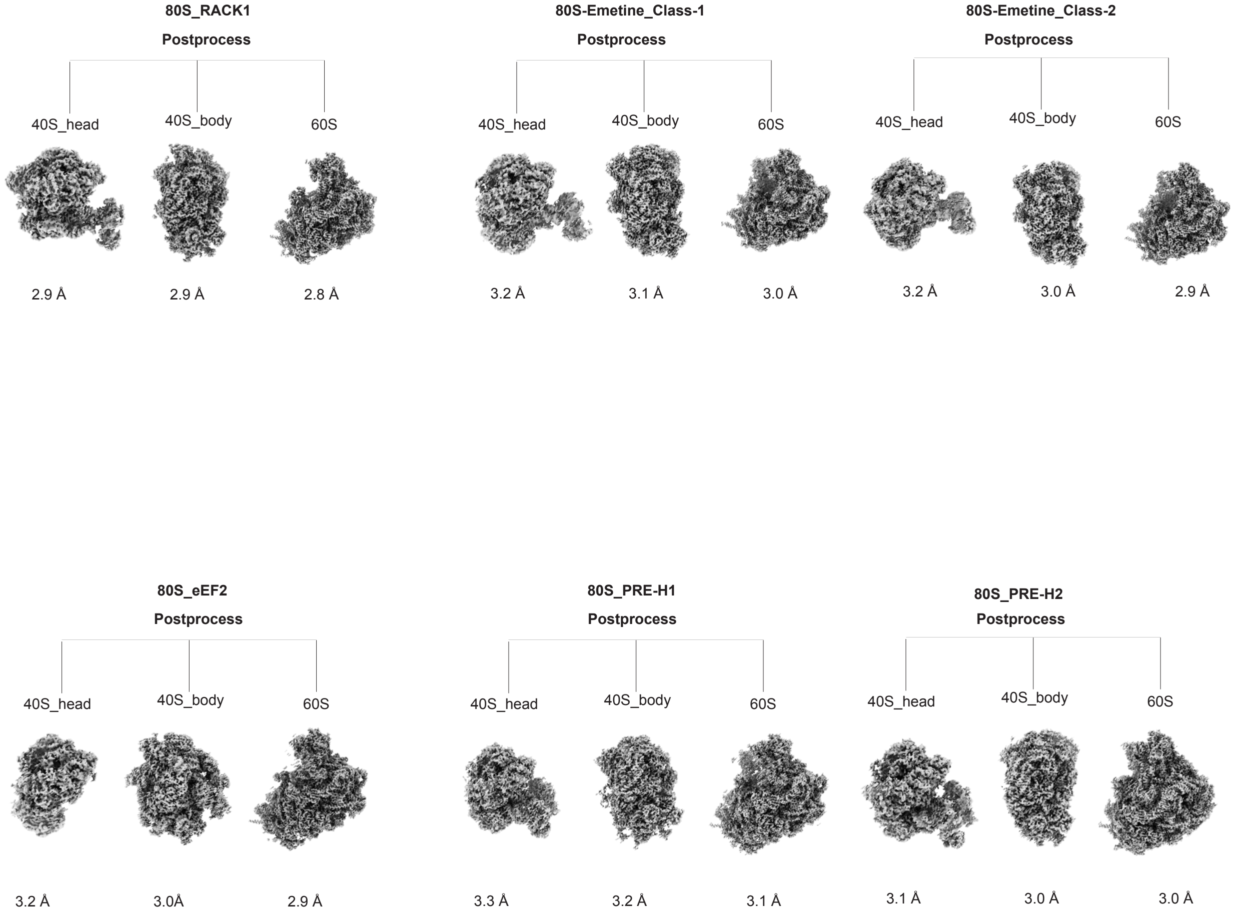
80S_RACK1
Postprocess
40S_head
40S_body
60S
2.9 Å
2.9 Å
2.8 Å
80S-Emetine_Class-1
Postprocess
40S_head
40S_body
60S
3.2 Å
3.1 Å
3.0 Å
80S-Emetine_Class-2
Postprocess
40S_head
40S_body
60S
3.2 Å
3.0 Å
2.9 Å
80S_eEF2
Postprocess
40S_head
40S_body
60S
3.2 Å
3.0Å
2.9 Å
80S_PRE-H1
Postprocess
40S_head
40S_body
60S
3.3 Å
3.2 Å
3.1 Å
80S_PRE-H2
Postprocess
40S_head
40S_body
60S
3.1 Å
3.0 Å
3.0 Å

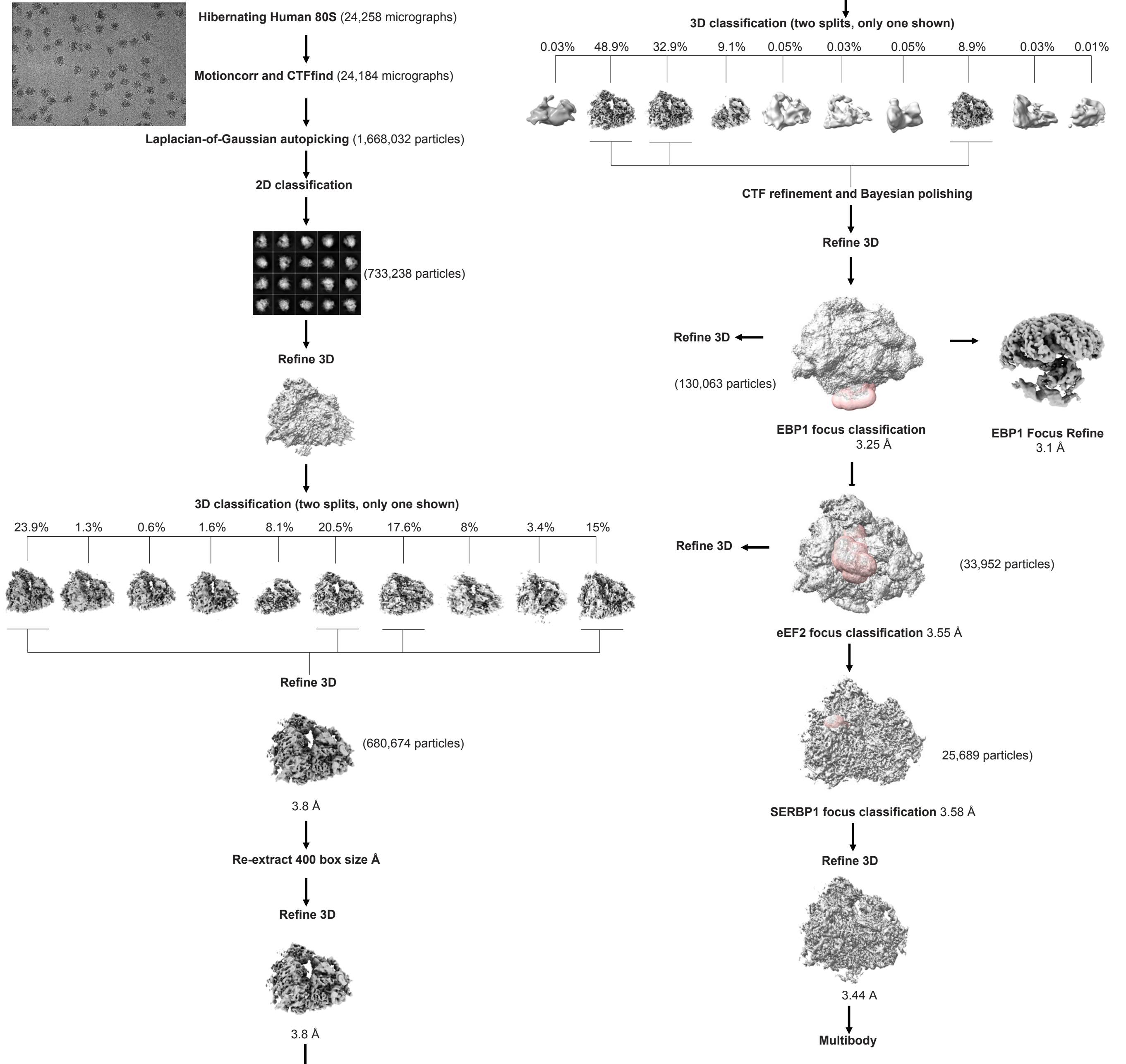

Hibernating Human 80S (24,258 micrographs)
Motioncorr and CTFfind (24,184 micrographs)
Laplacian-of-Gaussian autopicking (1,668,032 particles)
2D classification
(733,238 particles)
Refine 3D
3D classification (two splits, only one shown)
23.9%
1.3%
0.6%
1.6%
8.1%
20.5%
17.6%
8%
3.4%
15%
Refine 3D
(680,674 particles)
3.8 Å
Re-extract 400 box size Å
Refine 3D
3.8 Å
3D classification (two splits, only one shown)
0.03%
48.9%
32.9%
9.1%
0.05%
0.03%
0.05%
8.9%
0.03%
0.01%
CTF refinement and Bayesian polishing
Refine 3D
Refine 3D
(130,063 particles)
EBP1 focus classification
3.25 Å
EBP1 Focus Refine
3.1 Å
Refine 3D
(33,952 particles)
eEF2 focus classification 3.55 Å
25,689 particles)
SERBP1 focus classification 3.58 Å
Refine 3D
3.44 A
Multibody

Table S2: Cryo-EM data collection, refinement and validation statistics

	80S_RACK1_P-tRNA (EMDB- YYYY) (PDB XXXX)				80S_eEF2 (EMDB- YYYY) (PDB XXXX)			
**Data collection and processing**	40S_head	40S_body	60S	Composite	40S_head	40S_body	60S	Composite
Magnification	105,000	105,000	105,000	105,000	105,000	105,000	105,000	105,000
Voltage (kV)	300	300	300	300	300	300	300	300
Electron exposure (e–/Å^2)	1.0461	1.0461	1.0461	1.0461	1.0461	1.0461	1.0461	1.0461
Defocus range (μm)	-1.2 to -3.0	-1.2 to -3.0	-1.2 to -3.0	-1.2 to -3.0	-1.2 to -3.0	-1.2 to -3.0	-1.2 to -3.0	-1.2 to -3.0
Pixel size (Å)	0.832	0.832	0.832	0.832	0.832	0.832	0.832	0.832
Symmetry imposed	C1	C1	C1	C1	C1	C1	C1	C1
Initial particle images (no.)	6,364,045	6,364,045	6,364,045	6,364,045	6,364,045	6,364,045	6,364,045	6,364,045
Final particle images (no.)	24,661	24,661	24,661	24,661	58,749	58,749	58,749	58,749
Map resolution (Å)<br>FSC threshold	2.9<br>0.143	2.9<br>0.143	2.8<br>0.143	-	3.2<br>0.143	3.0<br>0.143	2.9<br>0.143	-
Map resolution range (Å)	2.5 to 5	2.5 to 5.0	2.5 to 4.5	-	2.5 to 6.5	2.5 to 6.5	2.5 to 4.5	-
**Refinement**								
Initial model used (PDB code)	5XXU	5XXU	5XXB	5XXU and 5XXB	5XXU	5XXU	5XXB	5XXU and 5XXB
Model resolution (Å)<br>FSC threshold	3.0<br>0.5	2.9<br>0.5	2.9<br>0.5	2.9<br>0.5	3.3<br>0.5	3.0<br>0.5	2.9<br>0.5	3.0<br>0.5
Model resolution range (Å)	2.4 to 3.0	2.4 to 2.9	2.3 to 2.9	1.6 to 2.9	2.8 to 3.3	2.7 to 3.0	2.2 to 2.9	1.7 to 3.0
Map sharpening *B* factor (Å^2)	-25	-25	-25	-25	-25	-25	-25	-25
Model composition<br>Non-hydrogen atoms<br>Protein residues<br>Nucleotide<br>Chains	 36,182<br>2,566<br>766<br>41	 51,872<br>3,086<br>1,282<br>34	 121,433<br>6,215<br>3,352<br>44	 194,133<br>10,851<br>5,054<br>89	 32,225<br>2,254<br>694<br>39	 56,809<br>3,843<br>1,236<br>32	 122,928<br>6,219<br>3,420<br>45	 194,244<br>11,388<br>4,972<br>84
B factors (Å^2)<br>Protein<br>Nucleotide	 91.96<br>70.17	 49.17<br>48.47	 37.63<br>35.73	 44.54<br>38.73	 53.61<br>70.74	 68.98<br>51.54	 38.30<br>38.42	 46.82<br>39.68
R.m.s. deviations								

Bond lengths (Å)<br>Bond angles (°)	0.005<br>0.684	0.003<br>0.575	0.006<br>0.713	0.006<br>1.013	0.003<br>0.656	0.003<br>0.611	0.004<br>0.662	0.005<br>0.831
Validation<br>MolProbity score<br>Clashscore<br>Rotamer outliers (%)	 1.95<br>10.00<br>0.00	 1.78<br>8.23<br>0.00	 1.75<br>8.25<br>0.00	 1.86<br>10.76<br>0.86	 2.00<br>13.77<br>0.06	 1.94<br>12.43<br>0.00	 1.90<br>10.10<br>0.02	 2.06<br>12.38<br>0.02
Ramachandran plot<br>Favored (%)<br>Allowed (%)<br>Outliers (%)	 93.41<br>6.43<br>0.16	 95.26<br>4.71<br>0.03	 95.61<br>4.34<br>0.05	 95.51<br>4.38<br>0.11	 94.87<br>5.04<br>0.09	 95.17<br>4.72<br>0.11	 95.53<br>5.40<br>0.07	 92.82<br>6.44<br>0.75

	PRE-H1<br>(EMDB- YYYY)<br>(PDB XXXX)				PRE-H2<br>(EMDB- YYYY)<br>(PDB XXXX)			
**Data collection and processing**	40S_head	40S_body	60S	Composite	40S_head	40S_body	60S	Composite
Magnification	105,000	105,000	105,000	105,000	105,000	105,000	105,000	105,000
Voltage (kV)	300	300	300	300	300	300	300	300
Electron exposure (e–/Å^2)	1.0461	1.0461	1.0461	1.0461	1.0461	1.0461	1.0461	1.0461
Defocus range (µm)	-1.2 to -3.0	-1.2 to -3.0	-1.2 to -3.0	-1.2 to -3.0	-1.2 to -3.0	-1.2 to -3.0	-1.2 to -3.0	-1.2 to -3.0
Pixel size (Å)	0.832	0.832	0.832	0.832	0.832	0.832	0.832	0.832
Symmetry imposed	C1	C1	C1	C1	C1	C1	C1	C1
Initial particle images (no.)	6,364,045	6,364,045	6,364,045	6,364,045	6,364,045	6,364,045	6,364,045	6,364,045
Final particle images (no.)	6,952	6,952	6,952	6,952	13,848	13,848	13,848	13,848
Map resolution (Å)<br>FSC threshold	3.3<br>0.143	3.2<br>0.143	3.1<br>0.143	-	3.1<br>0.143	3.0<br>0.143	3.0<br>0.143	-
Map resolution range (Å)	2.5 to 7.0	2.5 to 7.0	2.5 to 6.0	-	2.5 to 5.5	2.5 to 6.0	2.5 to 5.0	-
**Refinement**								
Initial model used (PDB code)	5XXU	5XXU	5XXB	5XXU and 5XXB	5XXU	5XXU	5XXB	5XXU and 5XXB
Model resolution (Å)<br>FSC threshold	3.3<br>0.5	3.2<br>0.5	3.1<br>0.5	3.0<br>0.5	3.1<br>0.5	3.0<br>0.5	3.0<br>0.5	2.9<br>0.5

Model resolution range (Å)	2.7 to 3.3	2.6 to 3.2	2.5 to 3.1	1.7 to 3.0	2.5 to 3.1	2.4 to 3.0	2.4 to 3.0	1.7 to 2.9
Map sharpening *B* factor (Å^2)	-25	-25	-25	-25	-25	-25	-25	-25
Model composition								
Non-hydrogen atoms	31,792	51,892	121,433	190,419	35,437	51,872	121,433	190,436
Protein residues	2,063	3,086	6,215	10,591	2,254	3,086	6,215	10,596
Nucleotide	747	1,283	3,352	4.973	844	1,282	3,352	4,973
Chains	49	34	44	88	41	34	44	82
B factors (Å^2)								
Protein	63.25	41.56	34.90	45.58	71.37	54.64	35.61	39.62
Nucleotide	64.41	35.73	36.18	41.33	63.30	46.96	34.41	35.65
R.m.s. deviations								
Bond lengths (Å)	0.002	0.006	0.004	0.004	0.004	0.008	0.007	0.008
Bond angles (°)	0.598	0.783	0.647	0.674	0.671	0.957	0.808	0.970
Validation								
MolProbity score	1.92	1.98	1.91	1.93	1.91	1.97	1.92	1.96
Clashscore	10.72	11.42	9.57	10.79	10.60	10.96	9.55	10.62
Rotamer outliers (%)	0.00	0.00	0.08	0.01	0.00	0.92	0.15	0.35
Ramachandran plot								
Favored (%)	94.55	93.96	93.96	94.37	94.68	93.73	93.69	93.79
Allowed (%)	5.40	5.91	6.03	5.55	5.13	6.24	6.12	6.05
Outliers (%)	0.05	0.13	0.02	0.09	0.18	0.03	0.18	0.15

	80S-Emetine_Class-1 (EMDB- YYYY) (PDB XXXX)				80S-Emetine_Class-2 (EMDB- YYYY) (PDB XXXX)			
**Data collection and processing**	40S_head	40S_body	60S	Composite	40S_head	40S_body	60S	Composite
Magnification	105,000	105,000	105,000	105,000	105,000	105,000	105,000	105,000
Voltage (kV)	300	300	300	300	300	300	300	300
Electron exposure (e–/Å^2)	1.0461	1.0461	1.0461	1.0461	1.0461	1.0461	1.0461	1.0461
Defocus range (μm)	-1.2 to -3.0	-1.2 to -3.0	-1.2 to -3.0	-1.2 to -3.0	-1.2 to -3.0	-1.2 to -3.0	-1.2 to -3.0	-1.2 to -3.0
Pixel size (Å)	0.832	0.832	0.832	0.832	0.832	0.832	0.832	0.832
Symmetry imposed	C1	C1	C1	C1	C1	C1	C1	C1

Initial particle images (no.)	6,364,045	6,364,045	6,364,045	6,364,045	6,364,045	6,364,045	6,364,045	6,364,045
Final particle images (no.)	15,598	15,598	15,598	15,598	47,159	47,159	47,159	47,159
Map resolution (Å)<br>FSC threshold	3.2<br>0.143	3.1<br>0.143	3.0<br>0.143	-	3.2<br>0.143	3.0<br>0.143	2.9<br>0.143	-
Map resolution range (Å)	2.5 to 6.0	2.5 to 6.0	2.5 to 6.5	-	2.5 to 5.5	2.5 to 5.5	2.5 to 4.5	-
**Refinement**								
Initial model used (PDB code)	5XXU	5XXU	5XXB	5XXU and 5XXB	5XXU	5XXU	5XXB	5XXU and 5XXB
Model resolution (Å)<br>FSC threshold	3.2<br>0.5	3.1<br>0.5	3.0<br>0.5	2.0<br>0.5	3.1<br>0.5	2.9<br>0.5	2.8<br>0.5	2.7<br>0.5
Model resolution range (Å)	2.7 to 3.2	2.7 to 3.1	2.6 to 3.0	1.7 to 2.9	2.4 to 3.1	2.4 to 2.9	2.2 to 2.8	1.6 to 2.7
Map sharpening *B* factor (Å^2)	-25	-25	-25	-25	-25	-25	-25	-25
Model composition<br>Non-hydrogen atoms<br>Protein residues<br>Nucleotide<br>Chains<br>Ligands	 23,899<br>1,525<br>572<br>17	 48,309<br>2,861<br>1,198<br>27	 121,433<br>6,215<br>3,352<br>44	 190,827<br>10,607<br>4,985<br>82<br>1	 22,763<br>1,563<br>501<br>16	 48,832<br>2,866<br>1,220<br>27	 122,776<br>6,295<br>3,388<br>45	 191,491<br>10,676<br>4,991<br>83<br>1
B factors (Å^2)<br>Protein<br>Nucleotide<br>Ligand	 57.59<br>51.16	 40.12<br>38.13	 31.70<br>31.82	 50.62<br>46.02<br>27.63	 41.85<br>46.86	 37.39<br>33.56	 19.88<br>21.44	 44.77<br>39.64<br>52.08
R.m.s. deviations<br>Bond lengths (Å)<br>Bond angles (°)	 0.003<br>0.717	 0.005<br>0.769	 0.007<br>0.772	 0.003<br>0.635	 0.006<br>0.868	 0.003<br>0.633	 0.004<br>0.658	 0.005<br>0.715
Validation<br>MolProbity score<br>Clashscore<br>Rotamer outliers (%)	 2.59<br>11.65<br>4.07	 2.42<br>8.84<br>3.91	 2.17<br>7.48<br>2.97	 2.00<br>10.28<br>0.01	 2.22<br>13.50<br>0.49	 1.91<br>8.15<br>0.16	 1.78<br>7.22<br>0.06	 1.95<br>9.86<br>0.16
Ramachandran plot<br>Favored (%)<br>Allowed (%)<br>Outliers (%)	 90.14<br>9.11<br>0.75	 91.65<br>7.24<br>1.11	 94.01<br>5.53<br>0.46	 92.54<br>7.01<br>0.45	 88.78<br>10.17<br>1.06	 92.61<br>6.43<br>0.96	 94.39<br>5.14<br>0.47	 93.36<br>5.96<br>0.68

	Human hibernating 80S<br>(EMDB- YYYY)<br>(PDB XXXX)			
**Data collection and processing**	40S_head	40S_body	60S	Composite
Magnification	105,000	105,000	105,000	105,000
Voltage (kV)	300	300	300	300
Electron exposure (e–/Å^2)	1.0461	1.0461	1.0461	1.0461
Defocus range (μm)	-1.2 to -3.0	-1.2 to -3.0	-1.2 to -3.0	-1.2 to -3.0
Pixel size (Å)	0.832	0.832	0.832	0.832
Symmetry imposed	C1	C1	C1	C1
Initial particle images (no.)	1,668,032	1,668,032	1,668,032	1,668,032
Final particle images (no.)	25,689	25,689	25,689	25,689
Map resolution (Å)<br>FSC threshold	4.3<br>0.143	3.9<br>0.143	3.3<br>0.143	-
Map resolution range (Å)	3.0 to 9.0	3.0 to 9.0	3.0 to 9.0	-
**Refinement**				
Initial model used (PDB code)	6Z6M	6Z6M	6Z6M and 6SXO	6Z6M and 6SXO
Model resolution (Å)<br>FSC threshold	4.7<br>0.5	4.1<br>0.5	3.2<br>0.5	3.2<br>0.5
Model resolution range (Å)	3.0 to 4.7	2.7 to 4.1	2.7 to 3.2	2.0 to 3.2
Map sharpening *B* factor (Å^2)	0	0	0	0
Model composition<br>Non-hydrogen atoms<br>Protein residues<br>Nucleotide<br>Chains	 37,399<br>2,338<br>881<br>23	 59,424<br>3,935<br>1,331<br>22	 141,820<br>6,872<br>4,048<br>46	 221,491<br>12,282<br>5,754<br>76

B factors (Å^2)				
Protein	149.73	88.05	62.96	92.10
Nucleotide	153.08	102.60	74.06	92.64
R.m.s. deviations				
Bond lengths (Å)	0.006	0.004	0.007	0.003
Bond angles (°)	0.859	0.724	0.778	0.632
Validation				
MolProbity score	2.38	2.65	2.30	2.01
Clashscore	17.67	12.97	9.19	10.57
Rotamer outliers (%)	0.05	4.54	2.69	0.15
Ramachandran plot				
Favored (%)	86.63	90.73	92.16	92.55
Allowed (%)	13.37	9.19	7.80	7.39
Outliers (%)	0.00	0.08	0.04	0.06
